# Engaging Underrepresented Older Adults in ADRD and Aging Research: A Scoping Review

**DOI:** 10.1093/geroni/igab046.3387

**Published:** 2021-12-17

**Authors:** Nisha Godbole, Jeannette Beasley, Simona Kwon, Timothy Roberts, Julie Kranick, Scott Sherman, Chau Trinh-Shevrin, Joshua Chodosh

**Affiliations:** 1 NYU Grossman School of Medicine, South Setauket, New York, United States; 2 NYU Grossman School of Medicine, New York, New York, United States; 3 NYU School of Medicine, New York, New York, United States; 4 NYU Langone Health, New York, New York, United States; 5 NYU Langone Medical Center, NYU Langone Medical Center, New York, United States

## Abstract

The rapidly aging and diversifying U.S. population coincides with increases in prevalence of Alzheimer’s disease and related dementias (ADRD) and other aging-related disorders. Unfortunately, older adults and racial and ethnic minorities are often underrepresented in research studies. The differing barriers that underrepresented older adults face in research engagement indicate that results from studies conducted on younger and majority populations may not maintain external validity outside of those groups. Therefore, efforts to engage diverse older adults in research is imperative. The goal of this scoping review was to summarize findings of the current state of National Institute on Aging (NIA) sponsored research, identifying extant literature on engaging diverse older adult populations in aging and ADRD research. Among 566 articles screened for inclusion, 436 were included in the final analysis. Results showed that African Americans were represented in over half the studies (63.5%), but Native Hawaiian/Pacific Islander and American Indian or Alaska Native populations were not well represented. Community- and convenience-based recruitment and retention strategies that have demonstrated prior success in research engagement were widely utilized. Racial, ethnic, and income status breakdowns were not included in 30.0%, 57.1%, and 53.4% of studies respectively, making it difficult to assess the applicability of findings for particular groups. Inclusion of Alzheimer’s disease patients or those with mild cognitive impairments was also poorly defined in most studies. Findings highlight gaps in existing literature that can be used to inform future research, and recruitment and retention strategies for engaging racial and ethnic minority older adults in research.

